# Supplementary figures and images for: Crystal structure of an RNA/DNA strand exchange junction
Source: PLoS One. 2022 Apr 18;17(4):e0263547. doi: 10.1371/journal.pone.0263547 (PMC9015157; doi:10.1371/journal.pone.0263547)

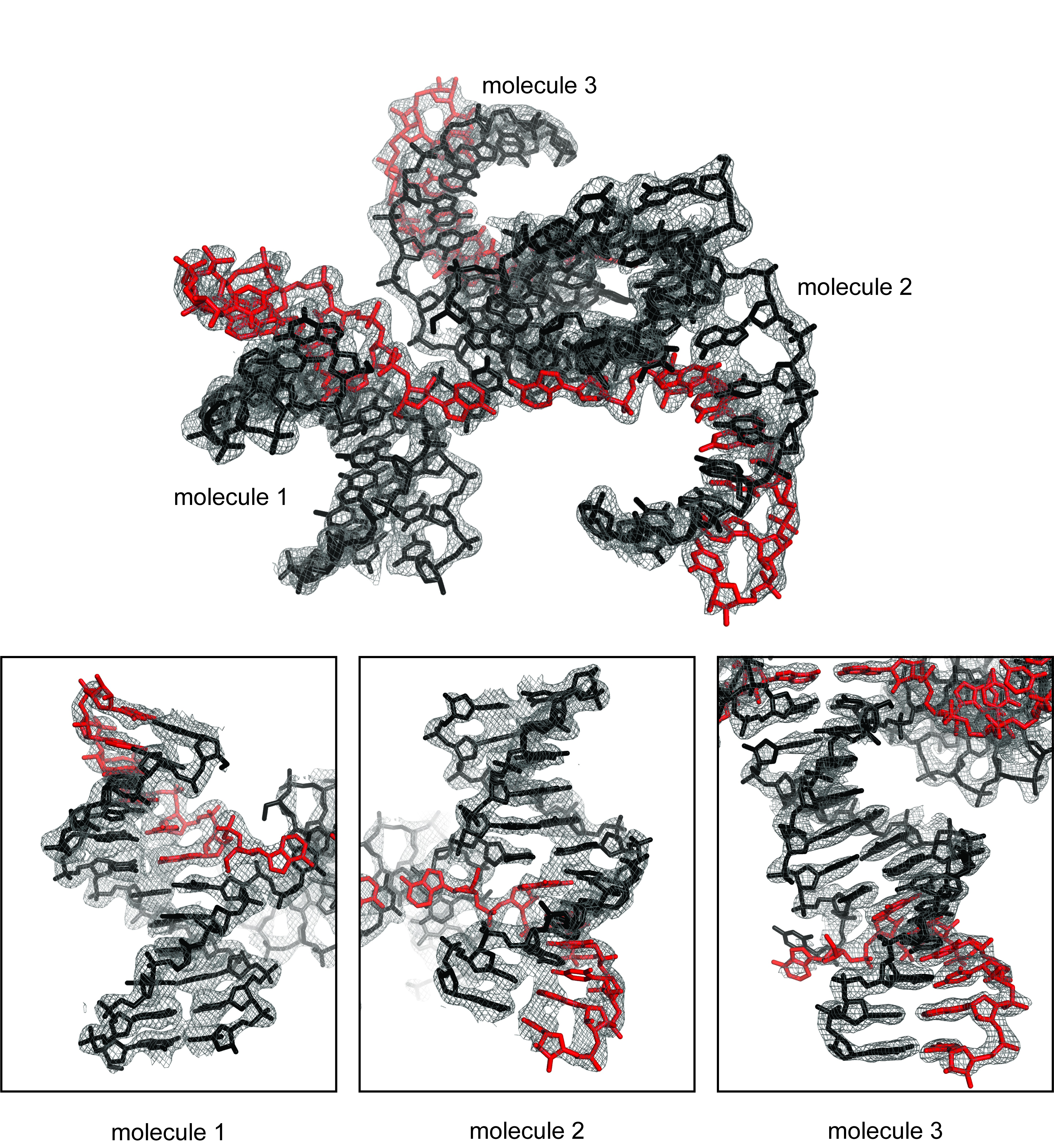

Supplement: S1 Fig — Model and composite omit 2mFo-DFc map (displayed at 1.5σ) of the asymmetric unit. Black, DNA; red, RNA. For clarity, the displayed density is truncated 2 Å from the atoms displayed in the model. “Blurriness” of the electron density is due to high atomic B-factors [53]. (TIF) [file pone.0263547.s001.tif]

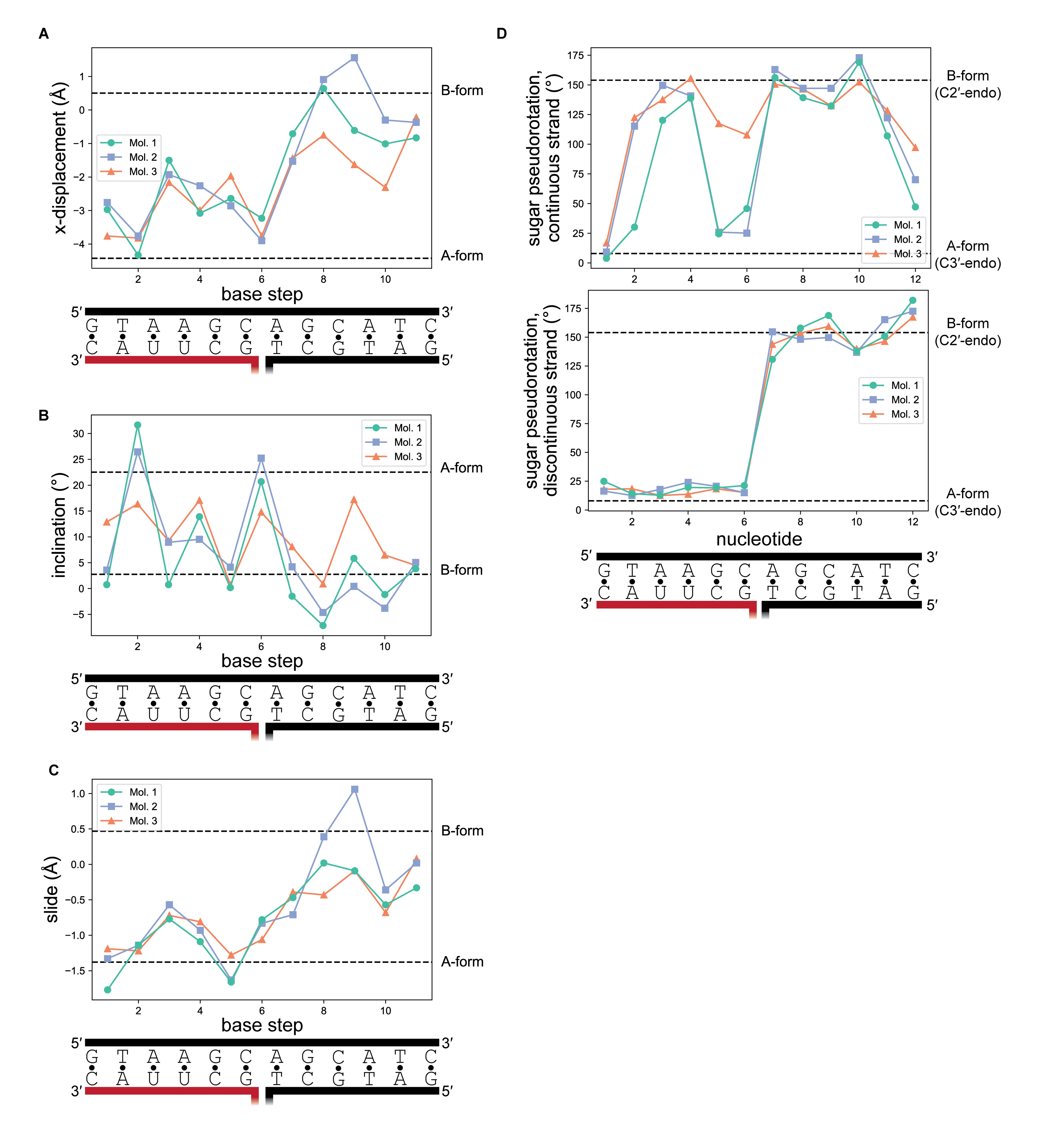

Supplement: S2 Fig — (A) X-displacement of the 11 base steps of the 12-bp helix. Black, DNA; red, RNA. (B) Inclination of the 11 base steps of the 12-bp helix. (C) Slide of the 11 base steps of the 12-bp helix. (D) Pseudorotation phase angles for the ribose/deoxyribose conformation at every nucleotide within the 12-bp helix (24 data points per molecule). The modeled sugar conformations might not be unique solutions for this dataset, as in many cases these structural details cannot be directly discerned from the 2mFo-DFc map. For this dataset, the most reliable parameters are those defined directly by the nucleobase and phosphate positions, which appear clearly in the 2mFo-DFc map (and likely impose indirect geometric constraints on the sugar pucker). (TIF) [file pone.0263547.s002.tif]

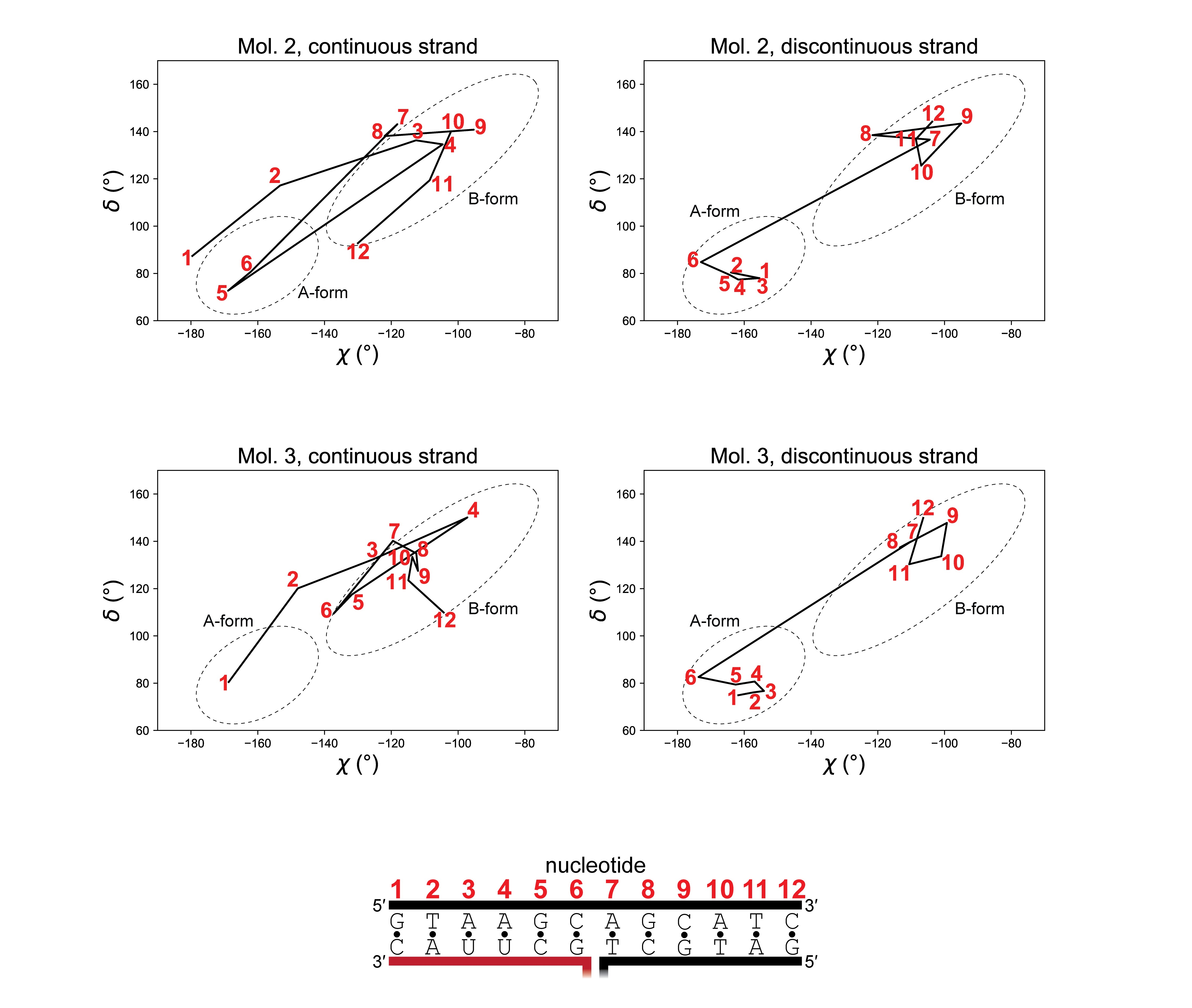

Supplement: S3 Fig — Analogous to Fig 4B. (TIF) [file pone.0263547.s003.tif]
