# Supplementary material for: Crystal structure of an RNA/DNA strand exchange junction
Source: PLoS One. 2022 Apr 18;17(4):e0263547. doi: 10.1371/journal.pone.0263547 (PMC9015157; doi:10.1371/journal.pone.0263547)
Supplement: Attachment — Submitted filename: 220301_response_to_reviewers.docx [file pone.0263547.s004.docx]

March 1, 2022

Petri Kursula, Ph.D.

Academic Editor

*PLOS ONE*

Re: PONE-D-22-01714

Dear Dr. Kursula,

We thank you and the referees for your comments and questions, which we have addressed below in **bold text**. In addition to those changes described below, we also removed a duplicate line that appeared in the legends to the Supporting Information.

Journal Requirements:

When submitting your revision, we need you to address these additional requirements.

1. Please ensure that your manuscript meets PLOS ONE's style requirements, including those for file naming. The PLOS ONE style templates can be found at

https://journals.plos.org/plosone/s/file?id=wjVg/PLOSOne_formatting_sample_main_body.pdf and

https://journals.plos.org/plosone/s/file?id=ba62/PLOSOne_formatting_sample_title_authors_affiliations.pdf

**We confirm that our manuscript meets PLOS ONE’s style requirements.**

2. Please review your reference list to ensure that it is complete and correct. If you have cited papers that have been retracted, please include the rationale for doing so in the manuscript text, or remove these references and replace them with relevant current references. Any changes to the reference list should be mentioned in the rebuttal letter that accompanies your revised manuscript. If you need to cite a retracted article, indicate the article’s retracted status in the References list and also include a citation and full reference for the retraction notice.

**We confirm that our reference list is complete and correct.**

3. Thank you for stating the following in the Competing Interests section:

"I have read the journal's policy and the authors of this manuscript have the following competing interests: The Regents of the University of California have patents issued and/or pending for CRISPR technologies on which G.J.K and J.A.D. are inventors. J.A.D. is a cofounder of Caribou Biosciences, Editas Medicine, Scribe Therapeutics, Intellia Therapeutics and Mammoth Biosciences. J.A.D. is a scientific advisor to Caribou Biosciences, Intellia Therapeutics, Scribe Therapeutics, Mammoth Biosciences, Algen Biotechnologies, Felix Biosciences and Inari. J.A.D. is a Director at Johnson & Johnson and at Tempus, and has research projects sponsored by Biogen and Apple Tree Partners."

Please confirm that this does not alter your adherence to all PLOS ONE policies on sharing data and materials, by including the following statement: "This does not alter our adherence to PLOS ONE policies on sharing data and materials.” (as detailed online in our guide for authors http://journals.plos.org/plosone/s/competing-interests). If there are restrictions on sharing of data and/or materials, please state these. Please note that we cannot proceed with consideration of your article until this information has been declared.

Please include your updated Competing Interests statement in your cover letter; we will change the online submission form on your behalf.

**We wish to amend our Competing Interests statement to read as follows:**

**“I have read the journal's policy and the authors of this manuscript have the following competing interests: The Regents of the University of California have patents issued and/or pending for CRISPR technologies on which G.J.K and J.A.D. are inventors. J.A.D. is a cofounder of Caribou Biosciences, Editas Medicine, Scribe Therapeutics, Intellia Therapeutics and Mammoth Biosciences. J.A.D. is a scientific advisor to Caribou Biosciences, Intellia Therapeutics, Scribe Therapeutics, Mammoth Biosciences, Algen Biotechnologies, Felix Biosciences and Inari. J.A.D. is a Director at Johnson & Johnson and at Tempus, and has research projects sponsored by Biogen and Apple Tree Partners. This does not alter our adherence to PLOS ONE policies on sharing data and materials.”**

Reviewer Comments

Reviewer #1: The paper describes a crystal structure in which a DNA oligomer makes complementary interactions with two shorter duplexes: one DNA and one RNA. Structures in which one of the complementary strands of a double DNA helix is replaced in part by RNA occur in some important biological processes. The question, from the structural point of view, is how the DNA, with its propensity to form B-type helices, accommodates RNA, which usually forms very different A-type helices. Of special interest in this case is the junction where the DNA-DNA duplex turns into DNA-RNA.

The science is sound, meticulously performed, the data have been carefully processed, the attached validation report is very neat. The paper is well written and illustrated. The results are presented concisely, the discussion is well balanced.

No PDB code is quoted in the paper but it is in the validation report and I have verified that the structural model is held waiting for publication.

**We have added the PDB code to Table 1.**

A minor point: some numbers are quoted to more decimal places than is probably reasonable. E.g. resolution range 35.335-1.637. Or RMSD values quoted to thousandths of an Angstrom. It's up to us to assess the accuracy of the values printed out by the software.

**We have rounded all numbers to a degree that better reflects the accuracy of the measurements/analyses.**

Reviewer #2: In the manuscript Cofsky and colleagues decribe a crystal structure of RNA/DNA complex that mimics a strand exchange function.The study is carefully carried out and structure is analyzed to fine detail. Descriptions on the methods are clear and authors disclose the problems also faced when interpreting the diffraction data, during reciprocal refinement and when interpreting the electron density maps affected by the anisotropy of the data. The coordinates, structure factors and raw images have been made available to the community (on hold for publication) and this is important especially in these problematic cases as it allows improvement of the software used in the field. Overall the analysis and writing is very careful and I have only minor suggestions to the authors how to improve the manuscript for the publication.

The data is very anisotropic and the authors describe this very clearly in the manuscript. I would however suggest that the authors would add a third resolution range to the data table which clearly indicates that the completeness is better than currently the numbers imply. This would, based on the description in the Methods section be [35.335 - 2.3 Å]. There is a slight discrepancy, as one would expect the ellipsoidal completeness to be higher than in the current table 1.

**We have adjusted Table 1 as suggested by the reviewer, and we include below the full completeness plots created by STARANISO for reviewer inspection. It is unclear to us why the ellipsoidal completeness begins to drop quickly before the I/σ(I)=1.2 cutoff (1.64 Å), but we believe that the updated Table 1, due to the adjustment suggested by the reviewer, now provides the reader with the key data necessary to interpret the general trends of completeness in our dataset.**

The authors use sharpened electron density maps in figure 1E (unsharpened comparison could be added in the supplement). However they describe well in the methods section that the density is not very clear for the different molecules. As careful analysis describing different variations in the A and B form helices is carried out, it would be good to add some representative overall electron density maps to the supplementary figures. As also the rmsd values were adjusted in the refinement to restrain the geometries this would allow better transparency for the reader.

**We have created a new supplementary figure (S1 Fig) depicting an overall electron density map, as suggested by the reviewer. We have changed the names of S1 Fig and S2 Fig to S2 Fig and S3 Fig, respectively. We have added a line to the methods (line 478 in the tracked-changes document) describing how we generated the new composite omit map.**

The high R-factor is slightly surprising for the resolution despite the anisotropy as authors also disclose. TLS refinement was carried out so analysis of the 15 TLS groups would be beneficial and could provide some insight to the molecule 3 disorder. The selected TLS segments are quite small. It would be slightly suprising if 7-mers and dissected pieces of 12-mers would not "move" in concert.

**The default output TLSMD images (see example of chain A, below) reveal that modeling the 12-mer as 3 groups involves combinations of “motions” that are not accessible to a rigid body (note, in particular, the tip of the pink part of the chain). Therefore, we believe this subdivision is likely justified. By considering known end-fraying, over/underwinding, and A/B transition motions (none of which can be modeled as rigid-body motions), we point out that non-rigid-body motions within this nucleic acid construct may not be so surprising after all.**

**However, while detailed discussion of local motions is beyond the scope of this work, we recognize the reviewer’s concern that extraneous TLS parameters may lead to overfitting. To assess this concern, we re-executed the final round of refinement with alternative TLS parameters, and we report the R-work/R-free values here:**

- **15 TLS groups (as in original manuscript): R-work / R-free = 0.237 / 0.284**
- **9 TLS groups (each chain): R-work / R-free = 0.240 / 0.283**
- **3 TLS groups (each molecule): R-work / R-free = 0.245 / 0.290**
- **No TLS refinement: R-work / R-free = 0.259 / 0.309**

**These trends do not suggest the occurrence of egregious overfitting. Additionally, the models and maps resulting from these refinements exhibited no obvious differences. Therefore, we believe that the models resulting from any of these refinements (including the one originally presented in the manuscript, which we have not changed) are acceptable and support the scientific conclusions pertinent to this manuscript.**

It is not entirely evident, when analyzing the structures, which features are resulting from crystal packing and which are expected interactions in a biological system. Solution data could be helpful, but authors could at least sharpen this discussion when discussing about intermolecular base pairing.

**Only intramolecular interactions should be considered for biological meaning because in known biological processes, it is not expected that two exchange junctions would ever contact each other in a way that resembles the crystal lattice. We have added the following sentences at line 244 (in the tracked-changes document) to clarify: “On the other hand, the flap conformations and the intermolecular base pairs observed here are peculiarities of the crystal lattice. During biological strand exchange processes, these overhung nucleotides would be unpaired and disordered [8].”**

Reviewer #3: Cofsky and co-workers present the crystal structure of a RNA/DNA hybrid strand exchange junction. Their structure sheds light on the geometrical properties of a RNA/DNA strand exchange junction showing a mixture of A-and B-form. This is a well described work that has been conducted thoroughly. I appreciate the illustration of the crystal lattice parameters and how the different molecules in the AU compare to each other in orientation and similarity. They go on with a detailed description of the geometric parameters resulting in some governing principles. They argue that their structure represents a key conformation especially for Cas9 genome editing, that can be useful in better modelling of cryo EM maps that have a much lower resolution.

I am genuinely happy with the presented results and the thoroughness of the analysis. However, I would appreciate more statistics on the model data like mean coordinate error and also an overall representation of the electron density fit. This would help to assess how accurate the model is in addition to the wealth of statistics provided by the authors.

**We have added Phenix’s estimate of the coordinate error to Table 1, as requested. We have added an overall representation of electron density fit as a supplementary figure, as described above.**
